# CD44 targeted delivery of oncolytic Newcastle disease virus encapsulated in thiolated chitosan for sustained release in cervical cancer: a targeted immunotherapy approach

**DOI:** 10.3389/fimmu.2023.1175535

**Published:** 2023-05-22

**Authors:** Kousain Kousar, Faiza Naseer, Maisa Siddiq Abduh, Sadia Anjum, Tahir Ahmad

**Affiliations:** ^1^ Industrial Biotechnology, Atta-Ur-Rahman School of Applied Biosciences, National University of Sciences and Technology, Islamabad, Pakistan; ^2^ Shifa College of Pharmaceutical Sciences, Shifa Tameer e Millat University, Islamabad, Pakistan; ^3^ Immune Responses in Different Diseases Research Group, Department of Medical Laboratory Sciences, Faculty of Applied Medical Sciences, King Abdulaziz University, Jeddah, Saudi Arabia; ^4^ Center of Excellence in Genomic Medicine Research, King Abdulaziz University, Jeddah, Saudi Arabia; ^5^ Department of Biology, University of Hail, Hail, Saudi Arabia

**Keywords:** oncolytic Newcastle disease virus, cervical cancer, green synthesis, sustained release, CD44, polymeric nanoparticles

## Abstract

**Introduction:**

Cervical cancer accounts for one of most common cancers among women of reproductive age. Oncolytic virotherapy has emerged as a promising immunotherapy modality but it comes with several drawbacks that include rapid clearance of virus from body due to immune-neutralization of virus in host. To overcome this, we encapsulated oncolytic Newcastle disease virus (NDV) in polymeric thiolated chitosan nanoparticles. For active targeting of virus loaded nanoformulation against CD44 (cluster of differentiation 44) receptors which are overly expressed on cancer cells, these nanoparticles were surface functionalized with hyaluronic acid (HA).

**Methods:**

Using half dose of NDV (TCID_50_ (50% tissue culture infective dose) single dose 3 × 10^5^), virus loaded nanoparticles were prepared by green synthesis approach through ionotropic gelation method. Zeta analysis was performed to analyse size and charge on nanoparticles. Nanoparticles (NPs) shape and size were analysed by SEM (scanning electron microscope) and TEM (transmission electron microscope) while functional group identification was done by FTIR (fourier transform infrared) and XRD (X-ray diffraction). Viral quantification was done by TCID_50_ and Multiplicity of infection (MOI) determination while oncolytic potential of NPs encapsulated virus was analysed by MTT (3-(4,5-dimethylthiazol-2-yl)-2,5-diphenyl tetrazolium bromide) assay and cell morphology analysis.

**Results:**

Zeta analysis showed that average size of NDV loaded thiolated chitosan nanoparticles surface functionalized with HA (HA-ThCs-NDV) was 290.4nm with zeta potential of 22.3 mV and 0.265 PDI (polydispersity index). SEM and TEM analysis showed smooth surface and spherical features of nanoparticles. FTIR and XRD confirmed the presence of characteristic functional groups and successful encapsulation of the virus. *In vitro* release showed continuous but sustained release of NDV for up to 48 hours. TCID_50_ for HA-ThCs-NDV nanoparticles was 2.63x 10^6^/mL titter and the nanoformulation exhibited high oncolytic potential in cell morphology analysis and MTT (3-(4,5-dimethylthiazol-2-yl)-2,5-diphenyl tetrazolium bromide) assay as compared to naked virus, in dose dependent manner.

**Discussion:**

These findings suggest that virus encapsulation in thiolated chitosan nanoparticles and surface functionalization with HA is not only helpful in achieving active targeting while masking virus from immune system but, it also gives sustained release of virus in tumor microenvironment for longer period of time that increases bioavailability of virus.

## 1 Introduction

Cervical cancer is one of the most common cancers among women and accounts for the highest mortality rate. According to a study on global burden of cervical cancer incidence and mortality, 604 127 new cases were reported in 2020 with 199,902 deaths in 2020 alone (1). Infection with Human papilloma virus is considered the major initiating factor but other reasons like high parity, smoking, genetic predisposition, multiple sexual partners, poor socio-economic status, sand early onset of sexual activity have been identified as major contributing factors for onset of cervical cancer (2). The conventional cervical cancer treatment approaches include chemotherapy, radiation therapy, complete surgical resection of tumor or the combination of these. All these approaches either have severe off target toxicities, adverse side effects or are highly invasive (3). Oncolytic virotherapy has emerged as a promising treatment modality against cancers and has the least undesirable effects in patients.

Oncolytic viruses (OV) are a group of viruses that have the ability to target and kill tumor cells. Their potent oncolytic activity is due to the fact that cancer cells have defected interferon pathway which does not enable them to effectively neutralize intracellular viruses as the normal cells do. Due to this deficient interferon pathway in cancer cells, OVs can continue to replicate until tumor cell apoptosis occurs and then the virus continues to infect the neighboring tumor cells. T-Vec (USA) and Onyx-015 (China) are the two FDA approved OVs used for the treatment of metastatic melanoma and head and neck carcinoma respectively. Presently, four OVs are approved for treatment of cancer in clinical setting, these include T-VEC, Oncorine (H101), Delytact and Rigvir. Delytact is a genetically engineered herpes simplex virus type 1 (HSV-1) that was approved by Japan in 2021 for the treatment of patients with malignant glioma (4). An avian influenza virus, Newcastle disease virus (NDV) has shown promising oncolytic potential in clinical research and is given preference over other viruses due to absence of pre-existing immunity against NDV in humans.

Newcastle disease virus belongs to Paramyxoviridae family of viruses and causes severe illness in birds. It is non-pathogenic in mammals. NDV is categorized into three pathotypes depending upon their pathogenicity namely, velogenic (virulent), mesogenic (intermediate) and lentogenic (avirulent). Its lentogenic strains Hitchner and LaSota are being used as live vaccines in poultry. In Glioma, NDV as oncolytic virus has completed phase I and II clinical trials (5). NDV stimulate apoptosis through both intrinsic and extrinsic pathways in tumor cell lines of ectodermal, endodermal and mesodermal origin. NDV infection in cancer cells can induce apoptosis by production of soluble TRAIL (TNF-related apoptosis-inducing ligand) and TNF-a (tumor necrosis factor-α) in specific manner by activation of initiator Caspase 8 (extrinsic pathway activator), while in some other cancer types oncolysis occurred without Caspase-8 activation (6). In addition, NDV kills tumor cells by disturbing mitochondrial membrane potential leading to the activation of Caspase-9 (intrinsic pathway activator) and through ER (endoplasmic reticulum) stress leading to activation of Caspase-12. Studies have suggested that NDV can induce potent systemic immune response that includes cellular, humoral, and mucosal immunity in humans. In human body, NDV is effectively neutralized by host immune system leading to early clearance of virus from the body. This emphasizes the need to enclose virus in a tumor targeted nano delivery system. A polymer-based, biocompatible drug delivery system such as chitosan or polyethylene glycol, is an ideal approach to protect virus from immune neutralization, for targeted delivery and sustained release of virus in tumor microenvironment (7).

Chitosan (Cs), which comes from partial deacetylation of the chitin, is a polysaccharide that possess ideal properties as a biocompatible drug delivery system. The structure of the chitosan can be physically modified due to presence of charged amine group (NH_2)_ (8). Thiolation of the chitosan by covalent crosslinking of thiol group (-SH group) with amine group of the chitosan improves its stability in polar environment and its mucoadhesive properties by enhancing targeted interaction at tumor site due to presence of covalently associated free thiol groups. In cancer therapy, thiolated chitosan (ThCs) have several favorable characteristics such as flexibility of surface functionalization with various targeting moieties, pH sensitive swelling behavior and controlled release of loaded therapeutic moiety. Furthermore, enhanced absorptive endocytosis of the drug/virus loaded thiolated chitosan nanocarriers occur due to formation of disulphide bonds with exofacial thiol groups of the transmembrane proteins (9, 10). To achieve active targeting, thiolated chitosan based drug delivery system can be surface functionalized with specific ligands (folic acid, hyaluronic acid) that can bind with specific receptors (folate receptor, CD44 receptor) on cancer cells.

Hyaluronic acid (HA) is a naturally occurring polysaccharide which bears negative charge, is hydrophilic in nature and consists of d-glucuronic acid and N-acetyl-d-glucosamine units. Its functional properties depend on its molecular weight and chain length which ranges from thousand to million Daltons. It can conjugate with various ligands, undergo conformational change and crosslink with bioactive compounds due to presence of N-acetyl groups, carboxylic acid groups, hydroxyl and glucuronic acid groups at functionally reactive sites (11). It has the ability to specifically bind with CD44 receptor which is overly expressed in many solid malignancies such as breast cancer, prostate cancer and cervical cancer.

CD44 (cluster of differentiation 44) receptor is also called H-CAM (homing cell adhesion molecule). Owing to its overexpression on solid tumors, hyaluronic acid modified polymers or hyaluronic acid itself could be used as a drug delivery system for targeted delivery in cancer (12). CD44 is involved in invasion, drug resistance and metastasis in cancer. Tumor cells greatly express this receptor as the CD44/HA interaction is indispensable for tumor invasion. In cervical cancer, high CD44 expression in HPV16 (human papillomavirus type 16) positive cell lines was associated with resistance to radiation therapy, high clonogenic capacity and advanced metastasis (13).

This study aims at formulation of NDV loaded thiolated chitosan nanoparticles, surface functionalized with HA for CD44 targeted delivery and sustained release of oncolytic NDV in cervical cancer cells. Ionotropic gelation method, which is based on electrostatic forces of attraction was used for the formulation of these nanoparticles and no acid/alkali or harsh chemicals were used during synthesis process. This encapsulation of virus in a targeted nano delivery system will increase its retention in tumor microenvironment, enhance viral uptake by tumor cells and increase bioavailability of virus due to mucoadhesive properties of thiolated chitosan. The graphical abstract in **Figure 1** shows the experimental design of current study.

### 1.1 Graphical abstract

**Figure 1:**
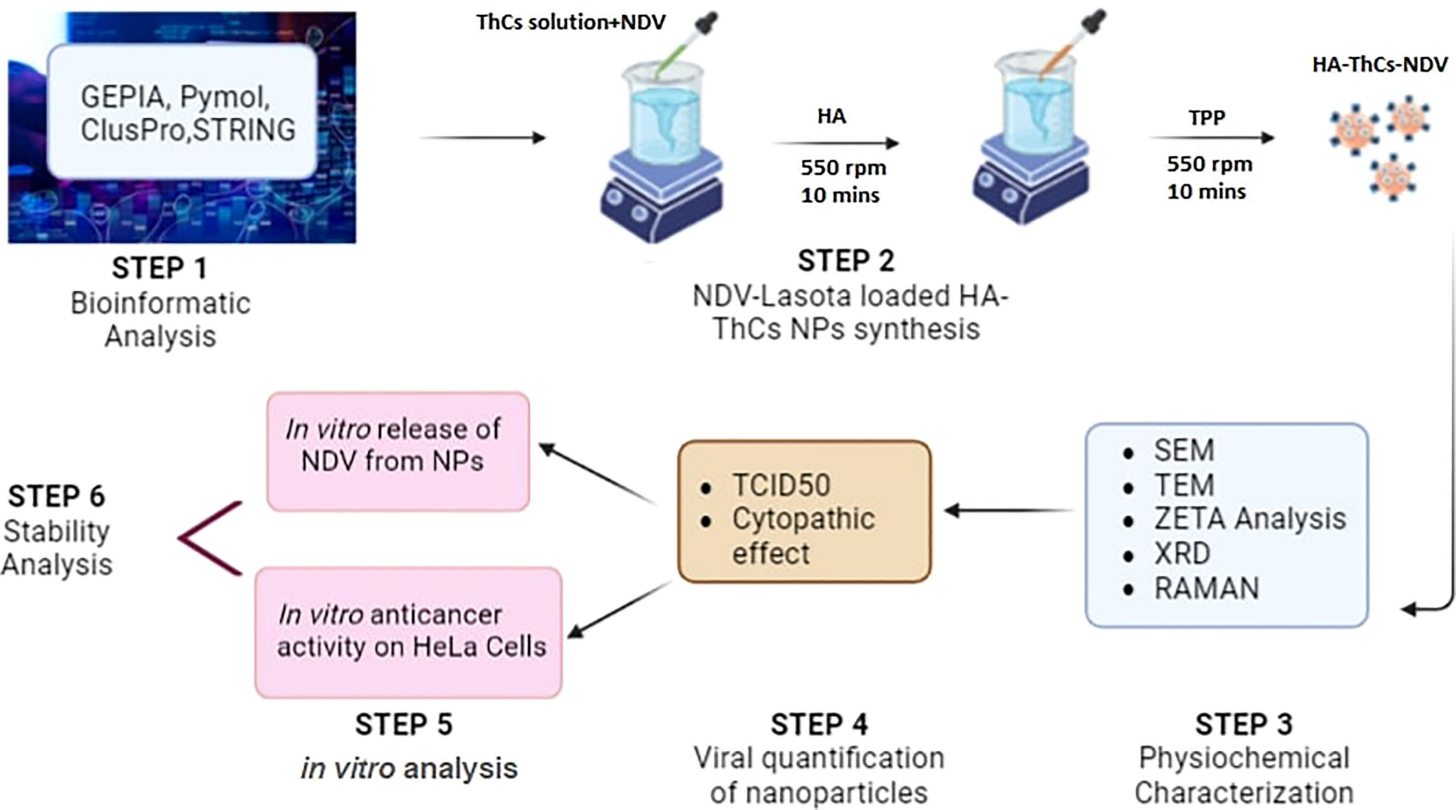
Graphical abstract of NDV loaded NPs synthesis, characterization and *in vitro* anticancer activity analysis.

## 2 Methodology

### 2.1 Materials

Tripolyphosphate polyanions (TPP), thiolated Chitosan (ThCs) 50-190 kilodaltons (low molecular weight) with 70%-80% deacetylation from Scharlau (Germany). Thioglycolic acid (TGA) (C_2_H_4_O_2_S), Sodium dihydrogen phosphate (NaH_2_PO_4_), Hydroxylamine (H_3_NO), Dipotassium hydrogen phosphate (K_2_HPO_4_), Sodium hydroxide (NaOH), Calcium chloride (CaCl_2_), Glacial acetic acid (CH_3_COOH) and Potassium dihydrogen phosphate (KH_2_PO_4_) were bought from Merck (Germany). High molecular weight hyaluronic acid 1500kd, Sodium borohydride (NaBH_4_), high retention dialysis membrane (12000–14,000Mw cut-off), and artificial mucin were ordered from Sigma-Aldrich USA and provided by Biolife Technologies and Science Home store, Islamabad, Pakistan. The Virology and immunology lab, Atta-ur-Rehman School of Applied Biosciences (ASAB), National University of Science and Technology (NUST), Islamabad, Pakistan provided distilled water, chemicals and solvents of analytical grade. Human cervical cell line, HeLa was cordially provided by Professor Saeed Khan, Dow University of Health Sciences, Ojha Campus.

### 2.2 Bioinformatic analysis

In order to evaluate the dynamics of CD44-HA binding, protein-protein interaction of CD44 with other proteins crucial to oncogenesis, interaction of NDV structural proteins with cellular counterparts and expression of CD44 receptor on HeLa cells, bioinformatic analysis was performed. GEPIA (gene expression profiling interactive analysis), a bioinformatic tool was used to analyze expression of CD44 on normal cervical and cervical cancer tissues. GEPIA works by extracting data from TCGA (The Cancer Genome Atlas) and GTEx (genotype-tissue expression) for cancer and normal tissues respectively. STRING (functional protein association networks) database was used to analyze physical and functional interaction of CD44 with its functional counterparts in human tissues. Data from Human Protein Atlas was used to check the expression of CD44 on human cervical cell line HeLa.

#### 2.2.1 Protein-protein interaction studies

To prove our hypothesis of CD44 targeted interaction, we performed *in silico* analysis of viral proteins and host cell proteins. For protein-protein interaction studies we used ClusPro and PyMOL. ClusPro 2.0 is one of the most commonly used protein-protein docking server which uses three different steps namely clustering on the basis of root mean square deviation (RMSD) of approximately 1000 lowest energy structures, rigid body docking and energy minimization in order to remove the steric clashes (14, 15). PyMOL (1.9 version) was used for the manual inspection of proteins (downloaded from PDB) and for the analysis of protein-protein interactions.

The viral targeted proteins were hemagglutinin, neuraminidase, viral fusion protein and matrix protein. Viral fusion protein and matrix protein are a type of glycoproteins that are the part of viral capsid while hemagglutinin act as the receptor binding site. The sequence for both the proteins was obtained from protein data bank (PDB.com) (PDB ID: 2RKC for Hemagglutinin and PDB ID: 5YXW for fusion protein) and were inspected manually by PyMOL.

The host cell proteins namely Tumor Necrosis Factor (TNF) (PDB ID: 1CA4), Cyclooxygenase II (COX-II) (PDB ID: 1CX2),NLR (Nod-like receptor) family pyrin domain containing 3 (NLRP3) (PDB ID: 7PZD) and NFkB (Nuclear factor kappa-light-chain-enhancer of activated B cells) (PDB ID: 1A3Q) were chosen for interaction studies (as shown in **Table 1**). these proteins were chosen due to the fact that they are most abundantly present in human cells and their interaction with viral proteins have the ability to stimulate the immune response which ultimately lead to tumor cell apoptosis (16, 17).

**Table 1:** The weighted energy scores for all host-viral protein interactions.

Serial No.	Receptor Protein	Ligand Protein	Weighted Energy Scores in KJ/mol	Obtained From
**1**	NFkB	NDV fusion protein	-824.1	ClusPro
**2**	NFkB	NDV hemagglutinin neuraminidase	-742.7	ClusPro
**3**	NFkB	NDV matrix protein	-584.4	ClusPro
**4**	TNF	NDV fusion protein	-848.2	ClusPro
**5**	TNF	NDV hemagglutinin neuraminidase	-955.2	ClusPro
**6**	TNF	NDV matrix protein	-800.7	ClusPro
**7**	NLRP3	NDV fusion protein	-787.2	ClusPro
**8**	NLRP3	NDV hemagglutinin neuraminidase	-849.9	ClusPro
**9**	NLRP3	NDV matrix protein	-720	ClusPro
**10**	COX II	NDV fusion protein	-1426	ClusPro
**11**	COX II	NDV hemagglutinin neuraminidase	-1185.6	ClusPro
**12**	COX II	NDV matrix protein	-845.8	ClusPro

### 2.3 Nanoparticle optimization via Box Behnken factorial design

For nanoparticle optimization and decreasing number and cost of experiments, central composite design was chosen by Design of Expert (DOE) version 8.0.6.1 following outputs given by Box Behnken factorial design. The dependent variables included zeta potential, polydispersity index (PDI) and size of nanoparticle. The concentration of poly-linker solution TPP was kept constant while the concentrations of ligand (HA), ThCs solution, and oncolytic Newcastle Disease Virus (NDV) were kept variable (18). **Table 2** shows the parameters and the outcomes from BoxBehnken factorial design

**Table 2:** Concentrations of variables, ligand (HA), polymer solution (ThCs), and oncolytic Newcastle Disease Virus (NDV) for the optimization of NDV-loaded NPs.

Run	A:HA conc	B:ThCs conc	C:NDV conc	Particle size	PDI	Zeta potentIal
**Unit**	mg	mg	ml	nm		+/-mV
**1**	2.75	10.00	0.50	463.9	0.363	16.4
**2**	0.50	1.00	0.30	416	0.584	-25.7
**3**	0.50	10.00	0.30	736.1	0.528	13.3
**4**	2.75	10.00	0.10	680.4	0.416	4.88
**5**	2.75	5.50	0.30	740	0.468	5.65
**6**	5.00	5.50	0.10	1683	1	-24
**7**	0.50	5.50	0.10	393.1	0.472	12
**8**	0.50	5.50	0.50	614	0.401	13.8
**9**	5.00	5.50	0.50	396.2	0.524	-25.6
**10**	5.00	1.00	0.30	275.6	0.372	11.5
**11**	2.75	5.50	0.30	339	0.363	18.1
**12**	2.75	1.00	0.10	438	0.678	-29.6
**13**	2.75	1.00	0.50	387.3	0.591	-23.7
**14**	5.00	10.00	0.30	752	1	28.5
**15**	2.75	5.50	0.30	358	0.4	-22

### 2.4 Propagation and purification of oncolytic Newcastle disease virus (NDV)

A stock solution of NDV at 1x10^16^ plaque forming unit (PFU) was cordially provided by University of Veterinary and Animal Sciences (UVAS), Lahore. This viral stock was extracted from Lasota strain, which is a naturally attenuated strain of oncolytic NDV. For purification of Lasota vaccine strain, it was propagated in allantoic cavity of embryonated chicken eggs for 120h post infection at 37°C. Following incubation, the allantoic cavity was harvested and centrifuged at 3000,5000 and 8000g/min at 4°C for 30 minutes. The supernatant was collected and further centrifuged at 30,000g/min at 4°C for 2.5 hours. Finally, the purified virus was resuspended in PBS (phosphate buffer saline) and stored at -20°C until further use (19, 20).

### 2.5 Preparation of ThCs polymer and crosslinker TPP solution

For optimized nanoformulation, ThCs solution was prepared in distilled water at concentration of 0.1%, a solution of TPP at concentration of 0.1mg/ml was dropwise added into ThCs solution while stirring constantly at 550rpm for 10 minutes to prepare nanoparticles by ionic gelation method and green synthesis approach. Once dissolved, dilution was prepared in distilled water and filtered through 0.22 mm to prepare a stock solution of 0.4% chitosan (w/v, 4 mg/ml) with a viscosity of 2.5460.1 centi Poise (cP) as measured using a Model DV-III plus Programmable Rheometer (Brookfield Engineering Laboratories, Middleboro, MA, USA). The resulting formulation was then sonicated by probe sonicator at 30mA for 2.5 minutes. Solution of HA at concentration of 0.5mg/ml was added later for surface functionalization. The resulting formulation was centrifuged, lyophilized, and stored at 4°C until further use (21).

### 2.6 Synthesis of NDV loaded HA-ThCs nanoformulation

To a solution of chitosan, half dose of NDV (TCID_50_ single dose 3 × 10^5^) was added drop wise while stirring constantly at 550rpm. After 10 minutes of stirring, TPP (0.1mg/ml) was added dropwise while stirring constantly at 550rpm for 15 minutes. The nanoformulation was then sonicated using probe sonicator. Following sonication, HA (0.5mg/ml) was added for surface coating while stirring constantly at magnetic stirrer for 10 minutes. Same protocol was used to prepare blank nanoparticles, excluding the virus addition step. The resulting formulation was centrifuged, lyophilized, and stored at 4°C until required (12, 22).

### 2.7 NDV-encapsulated nanoparticle characterization

#### 2.7.1 Physiochemical and morphological properties of NFs

To analyze the therapeutic efficacy and targeting potential of NDV-encapsulated nanoformulation at tumor site keeping in view the dynamics of tumor microenvironment, several properties of NPs were analyzed. Zeta analysis was used to assess the size, PDI and zeta potential of nanoparticles. Scanning electron microscopy (SEM), transmission electron microscopy (TEM) and RAMAN spectroscopy analysis was used to analyze the shape, surface morphology of NPs and vibrational modes of nanoparticles respectively. Fourier-transform infrared spectroscopy and X-ray diffraction (XRD) were used to explore functional groups (22). All assays were performed according to the mentioned protocols in our previous study (23).

### 2.8 NDV quantification in NPs

Three methods were used to quantify the number of viral particles encapsulated in nanoformulation.

#### 2.8.1 Viral plaque assay

In NPs, NDV titter was measured by using plaque assay. For this purpose, confluent monolayer of HeLa cells grown in DMEM in 24 well plate, cultured at 37°C and 5% CO_2_ were inoculated with different dilutions of virus and NDV nanoformulation (0.1-10 µL per well). After 1 hour contact time, the media was aspirated from wells, cells were replenished with DMEM + 5% FBS (fetal bovine serum) and 2% methylcellulose and left to incubate at 37°C and 5% CO_2_ for 5-7 days. After 7 days, the media was removed carefully, and cells were washed with PBS and were fixed with 4% paraformaldehyde (PFA). After 20 minutes incubation with PFA, cells were rewashed with PBS and stained using crystal violet. Manual counting was carried out for the quantification of viral plaques, and data is shown as mean ± SD (standard deviation) of three independent experiments performed (24).

#### 2.8.2 TCID_50_

The viral titter was measured by calculation of TCID_50_, following Reed and Munich Protocol (25). From 1:10 titer solution of lyophilized NDV NPs, dilutions ranging from 10^-2^ to 10^-11^ were prepared in 10 μL PBS at pH 7.2. Confluent monolayer of HeLa cells seeded at density of 0.7×10^4^ in 96 wells were inoculated with 10X serial dilution of NDV encapsulated NPs and placed in incubator at 37°C and 5% CO_2_ incubation. Final quantification of infected and uninfected wells was done on day 7 of post infection (p.i)d and the resulting titter was reported as TCID_50_/ml of HA-ThCs-NDV NPs. TCID_50_ determines the 50% infectious viral particle present in 1 ml of the sample. HeLa cells treated with pure NDV served as positive control while cells with only growth media served as negative control (26).

#### 2.8.3 Cytopathic effects (CPE)

Cytopathic effect (CPE) of HA-ThCs-NDV NPs on HeLa cells was determined by observing morphological changes in cells following treatment with NPs in dose and time dependent manner. CPE of NDV loaded nanoparticles on HeLa cells was compared with that of pure NDV at different dilutions in a 6 well plate. After 24 hours incubation, cells were washed with PBS, replenished with growth medium and left for incubation at 37°C and 5% CO_2._ At 7 p.i.d (post infection day), CPE was analyzed by observing morphological changes such as disaggregation, rounding, clumping and blobbing etc., by using inverted phase contrast microscopy (TCM-400, OEM-Optical, Labomed, USA) (19).

#### 2.8.4 Multiplicity of Infection (M.O.I.)

HeLa cells were seeded in a 6 well plate at seeding density of 2×10^6^ cells/10cm^2^. After 24 hours, the confluent monolayer was inoculated with HA-ThCs-NDV and pure NDV (Lasota) at MOI of 0.6, 1,5,8,12, 18 and 20. Cells were incubated with virus for 2 hours, followed by washing with DMEM media to remove unattached virus. Cells were then incubated at 37°C and 5% CO_2_ for 3 days (27).

### 2.9 *In vitro* release of virus from NPs

0.1g of lyophilized HA-ThCs-NDV nanoformulation was poured in a dialyzing membrane immersed in 50ml PBS (pH 7.2). The dissolution assembly was kept at 37°C on a magnetic hot plate with 100rpm. After specified time intervals of 0, 0.5, 1, 2, 4, 8, 10, 12, 24, 36, and 48 hours, 1.5 ml of sample was collected and centrifuged at 10,000g/min. All collected samples were analyzed on spectrophotometer at 595nm to analyze the release of NDV from nanoparticles. The time duration for release was plotted at X-axis while accumulative amount of virus released was plotted at Y-axis to get NDV release curve (28).

### 2.10 *In vitro* anticancer activity

*In vitro* anticancer activity of HA-ThCs-NDV was analyzed on HeLa cells which was derived from cervical cancer cells of a 31 year old African American women, Henrietta Lacks. These cells have multiple integrated copies of Human Papilloma virus-18 (HPV-18). One of the most common HPV type associated with HPV induced carcinogenesis (29).

#### 2.10.1 *In vitro* experimental groups

Three groups were formed for *in-vitro* experiment:

Group 1: Untreated control HeLa cells in DMEM medium.
Group 2: NDV treated HeLa cells.
Group 3: NDV loaded HA-ThCs NPs treated HeLa cells.

#### 2.10.2 MTT cell viability assay

Cytotoxicity potential of HA-ThCs-NDV and NDV was analyzed following published protocol with few amendments. HeLa cells were cultured in DMEM growth media, supplemented with 10% FBS and 500μL of Penicillin Streptomycin (pen strep) at seeding density of 1x10^4^ in 96 well plate and incubated at 37°C with 95% air and 5% CO_2_ until the cells formed confluent monolayer. The cells were used in their exponential growth phase. The experiment consisted of two groups. Group 1: Pure NDV Lasota strain (positive control) and Group 2: HA-ThCs-NDV, while cells with only growth media served as negative control. Cells were inoculated with NDV encapsulated NPs at MOIs (0.1, 0.6, 1, 5, 8, 12, 18 and 20) as compared with pure NDV Lasota strain on HeLa cells. Following 72 hours incubation period, MTT assay was performed. TCID_50_ was determined as proportion of the viable cells after treatment with NDV as compared to the control cells that were not infected with the virus (4).

### 2.11 Stability parameters

After period of 3 months, the stability associated parameters like surface morphology and zeta potential of HA-ThCs-NDV NPs were analyzed while the formulation was stored at conditions of 4°C and 37°C.

### 2.12 Statistical studies

All results from experiments were statistically analyzed using one-way analysis of variance (ANOVA) and student t-test with a significant P ≤ 0.05 with mean value of multiple readings and standard deviation (mean ± SD). All studies were conducted in triplicates.

## 3 Results

### 3.1 Bioinformatics analysis

**Figure 2:**
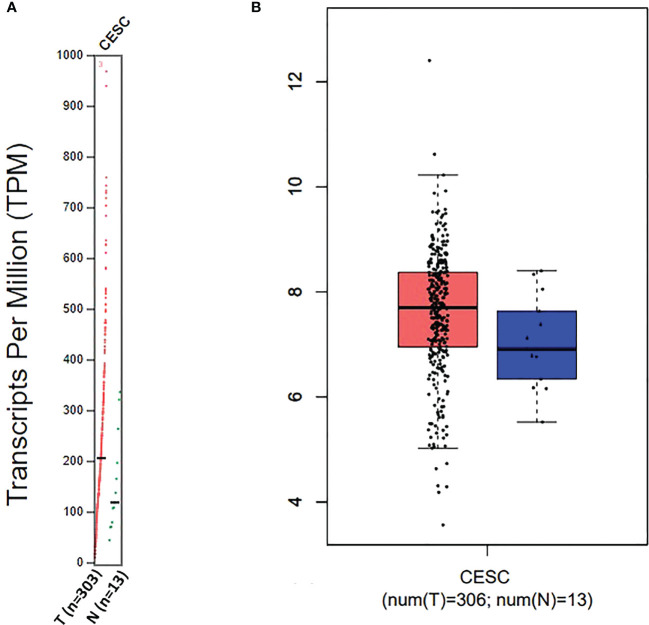
Dot plot **(A)** and Box plot **(B)** data for CD44 expression in Cervical squamous cell carcinoma and endocervical adenocarcinoma (CESC).

**Figure 3:**
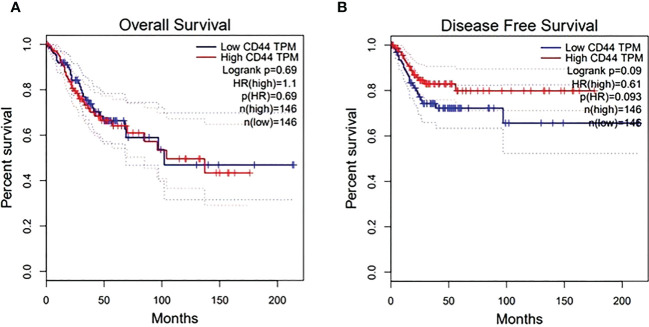
Effect of CD44 expression on overall surival **(A)** and disease free survival after treatment **(B)**. X-axis shows survival time in months while Y-axis shows the percent survival of patients.

**Figure 4:**
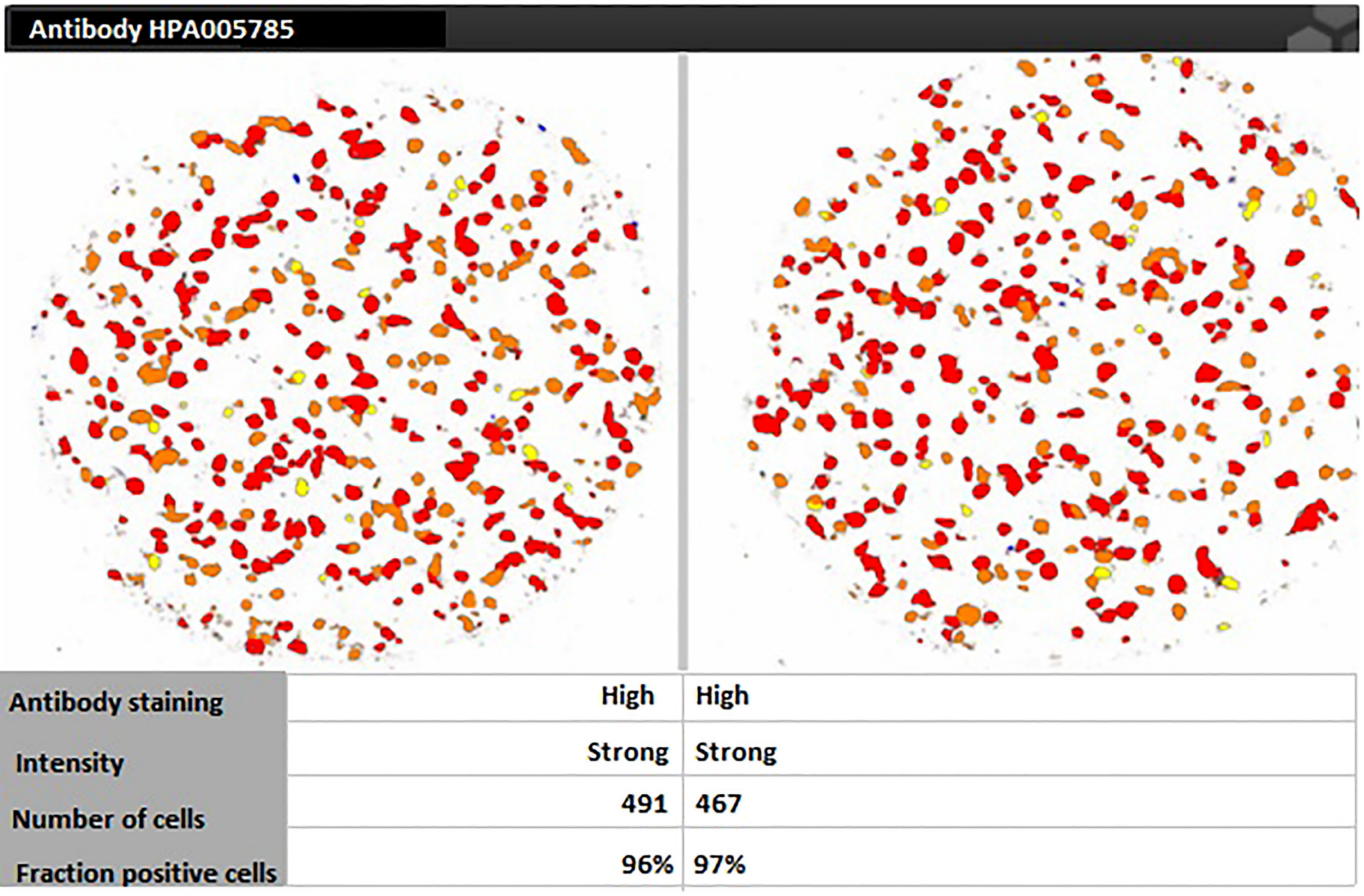
Human Protein Atlas immunohistochemical staining results indicating strong immunoreactivity of CD44 antibody HPA005785 with HeLa cells.

**Figure 5:**
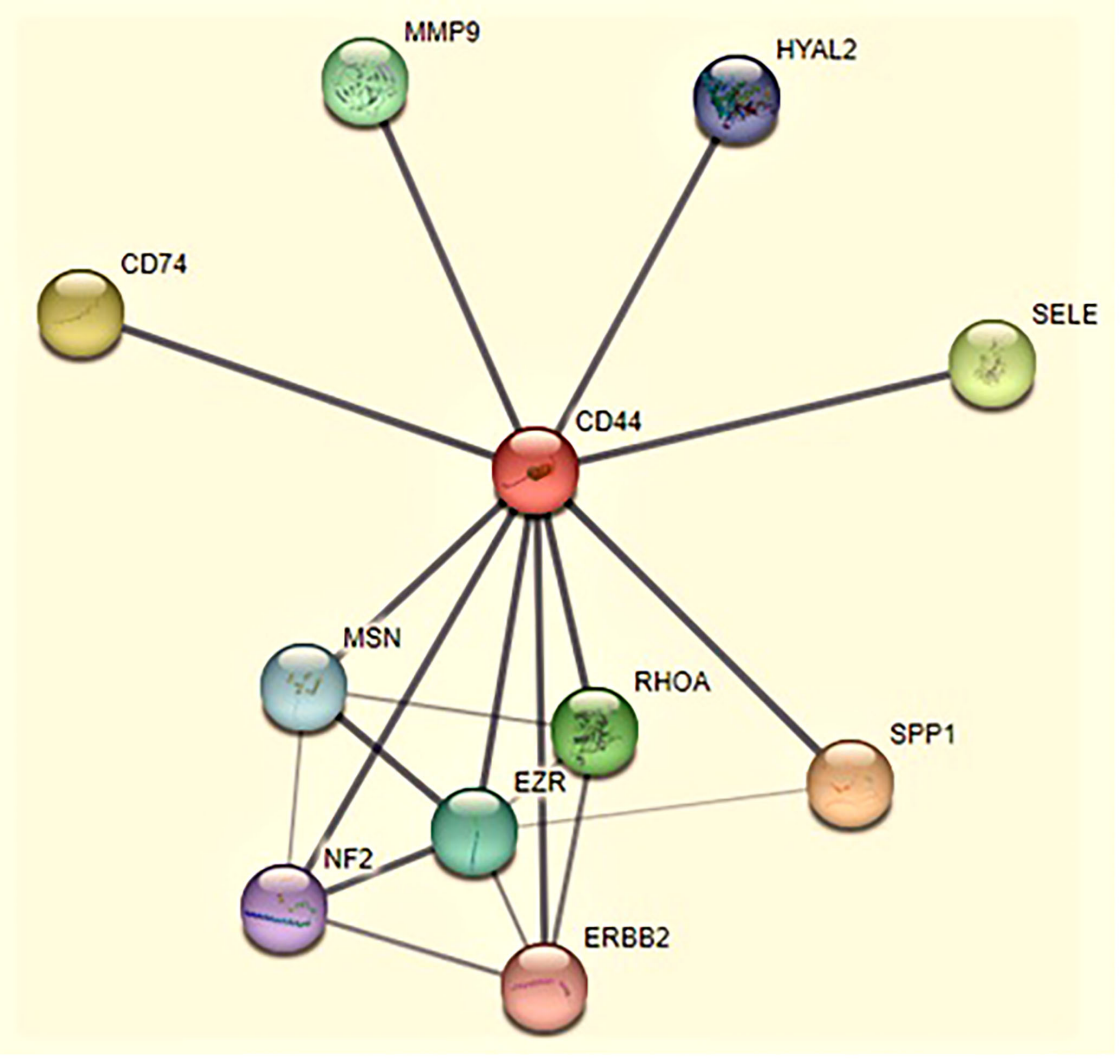
The interaction of CD44 with its functional counterparts in human during cancer. Line thickness indicates the strength of data support in this protein cluster. Color indicates different functional partners of CD44 that take part in tumorigenic activities such as SPP1 (secreted phosphoprotein 1) mediates cell-matrix interactions (light pink), CD74 (cluster of differentiation 74) regulates MHC (major histocompatibility complex) class II antigen processing (yellow), SELE (selectin E) regulates immune-adhesion and capillary morphogenesis (light green), RhoA (Ras homolog family member A) regulates cell polarity, shape, adhesion and locomotion by controlling actin polymerization (green). MMP9 (matrix metalloproteinases) cleaves extracellular matrix (ECM) protein to modulate invasion and metastasis (turquoise), EZR (ezrin) regulates adhesion signal pathways (blue), MSN (moesin) modulates cell-cell recognition and cell movement (sky blue), HYAL2 (hyaluronidase 2) have role in cell differentiation, proliferation and migration (dark blue), NF2 (neurofibromatosis type 2) modulates differentiation and apoptosis (indigo), ERBB2 (erythroblastic oncogene B) modulates tumorigenic transformation and chemoresistance (pink).

For targeting nanoparticles against CD44 receptor, analysis of its expression on targeted cells and role in tumorigenesis is important. The bioinformatics analysis showed a high expression of CD44 on cervical squamous cell carcinoma and endocervical adenocarcinoma (CESC) tissues as compared to normal cervical tissue after comparing tumor tissue data from TCGA and normal tissue data from GTEx. Dot plot (A) and Box plot (B) in **Figure 2** shows a substantial over expression of CD44 in cervical tumor tissues as compared to normal cervical tissues. Role of CD44 in overall survival and disease free survival is shown in **Figure 3**, which depicts that CD44 does not have significant role in overall survival but its high expression in tumor tissue strongly correlates with disease free survival in cervical cancer patients. Immunohistochemical staining data from Human Protein Atlas shows a strong immunoreactivity of CD44 antibody HPA005785 with HeLa cells as in **Figure 4**, orange colored cells indicate moderate/strong and red indicates very strong immunoreactivity. STRING analysis (**Figure 5**) shows the interaction of CD44 with its functional counterparts in human which associates with it physically or functionally during carcinogenesis.

### 3.2 Protein-protein docking

The protein-protein docking studies were performed for several different proteins of interest but only the most significant interactions are discussed below.

#### 3.2.1 Protein-protein interaction between viral fusion protein and host cell NFkB

**Figure 6:**
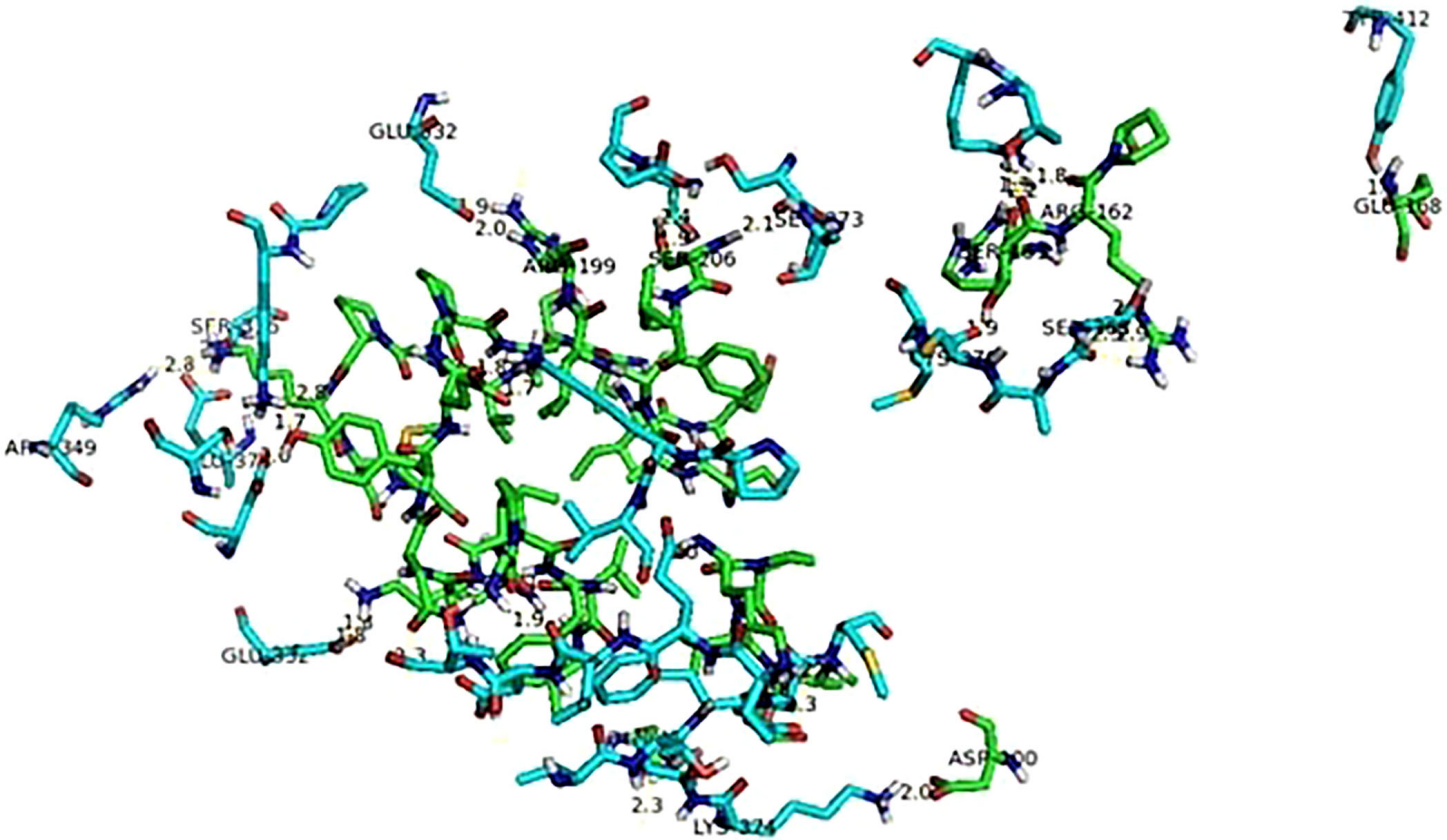
Interaction between NFkB (green sticks) and viral fusion protein (blue sticks).

The protein-protein interactions (**Table 1**) exhibited only polar contacts between the proteins. The type of polar interaction included both hydrogen bonding and ionic interactions. There were a total of 18 polar contact points between the viral fusion and NFƙB proteins. All of the contact point interaction distances are within the acceptable range (4 angstrom (Å) cutoff) which indicates the strong bonding between the proteins. There could be a lot of hydrophobic interaction among residues of both proteins making the interaction between proteins more stable and stronger. The amino acid interactions along with the distances are shown in **Table 3**. **Figure 6** shows interaction between NFkB and Viral fusion protein.

**Table 3:** Receptor and ligand protein amino acid interactions and distances.

Serial No	Amino acid (Receptor protein)	Amino acid (Ligand protein)	Distance in Angstrom	Type of interaction
**1**	GLU-168	TYR-412	1.9	Polar
**2**	LYS-127	GLU-376	2.8	Polar
**3**	LYS-127	SER-316	2.7	Polar
**4**	LYS-81	GLU-332	1.8, 1.8	Polar
**5**	ARG-162	SER-368	2.5, 2.1	Polar
**6**	ASP-100	LYS-374	2.0	Polar
**7**	SER-161	CYS-370	1.9	Polar
**8**	ARG-199	GLU-332	1.9, 2.0	Polar
**9**	LYS-127	SER-316	2.7	Polar
**10**	LYS-127	GLU-376	2.7	Polar
**11**	LYS-127	ARG-349	2.8	Polar
**12**	ARG-199	GLU-332	1.9, 2.0	Polar
**13**	SER-206	SER-373	2.1	Polar
**14**	SER-206	GLU-376	2.4	Polar

#### 3.2.2 Protein-protein interaction between human TNF and viral hemagglutinin neuraminidase

**Figure 7:**
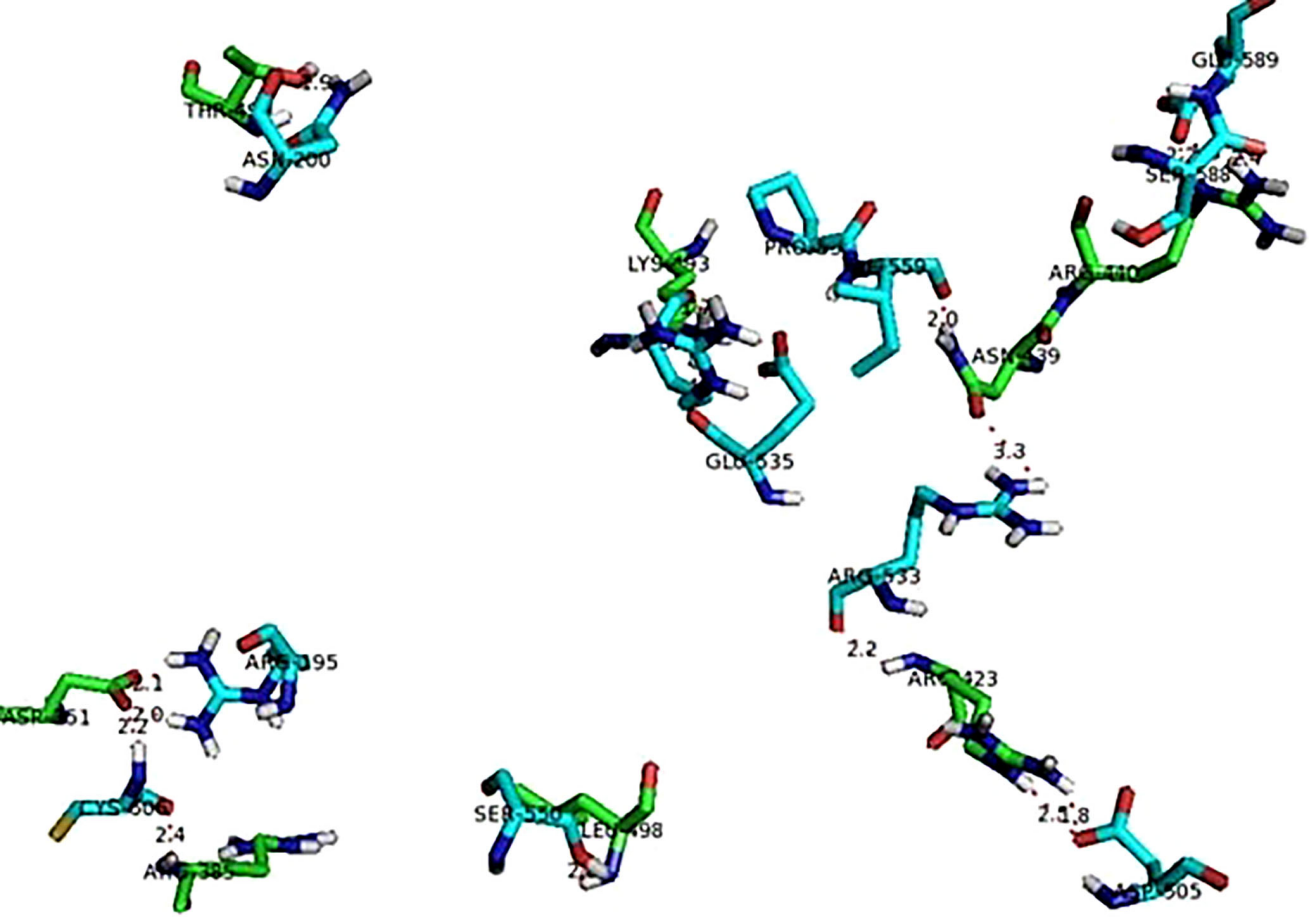
Interaction between TNF (green sticks) and viral hemagglutinin neuraminidase (blue sticks).

The protein-protein interactions showed only polar contacts between the proteins (**Table 4**). Several of the arginine residues were involved in both hydrogen bonding and ionic interaction. There were a total of 14 polar contact points between the human TNF and viral hemagglutinin neuraminidase proteins. All contact point interaction distances are within the acceptable range (4 Å) which indicates the strong bonding between the proteins. **Figure 7** shows interaction between human TNF and viral hemagglutinin neuraminidase.

**Table 4:** The type of receptor and ligand protein amino acid interactions and distances.

Serial No.	Amino acid (Receptor protein)	Amino acid (Ligand protein)	Distance in Angstrom	Type of interaction
**1**	THR-499	ASN-200	1.9	Polar
**2**	ASP-351	ARG-195	2.0, 2.1	Polar
**3**	ASP-351	CYS-606	2.2	Polar
**4**	ARG-385	CYS-606	2.4	Polar
**5**	LYS-493	ARG-556	1.7	Polar
**6**	LYS-493	GLU-535	1.7	Polar
**7**	ASN-439	ILE-559	2.0	Polar
**8**	LEU-498	SER-550	2.8	Polar
**9**	ARG-423	ASP-505	2.5, 1.8	Polar
**10**	ARG-440	GLU-389	1.8	Polar
**11**	ARG-440	SER-588	2.4	Polar
**12**	ASN-439	ARG-533	3.3	Polar

#### 3.2.3 Protein-protein interaction between human NLRP3 and viral fusion

**Figure 8:**
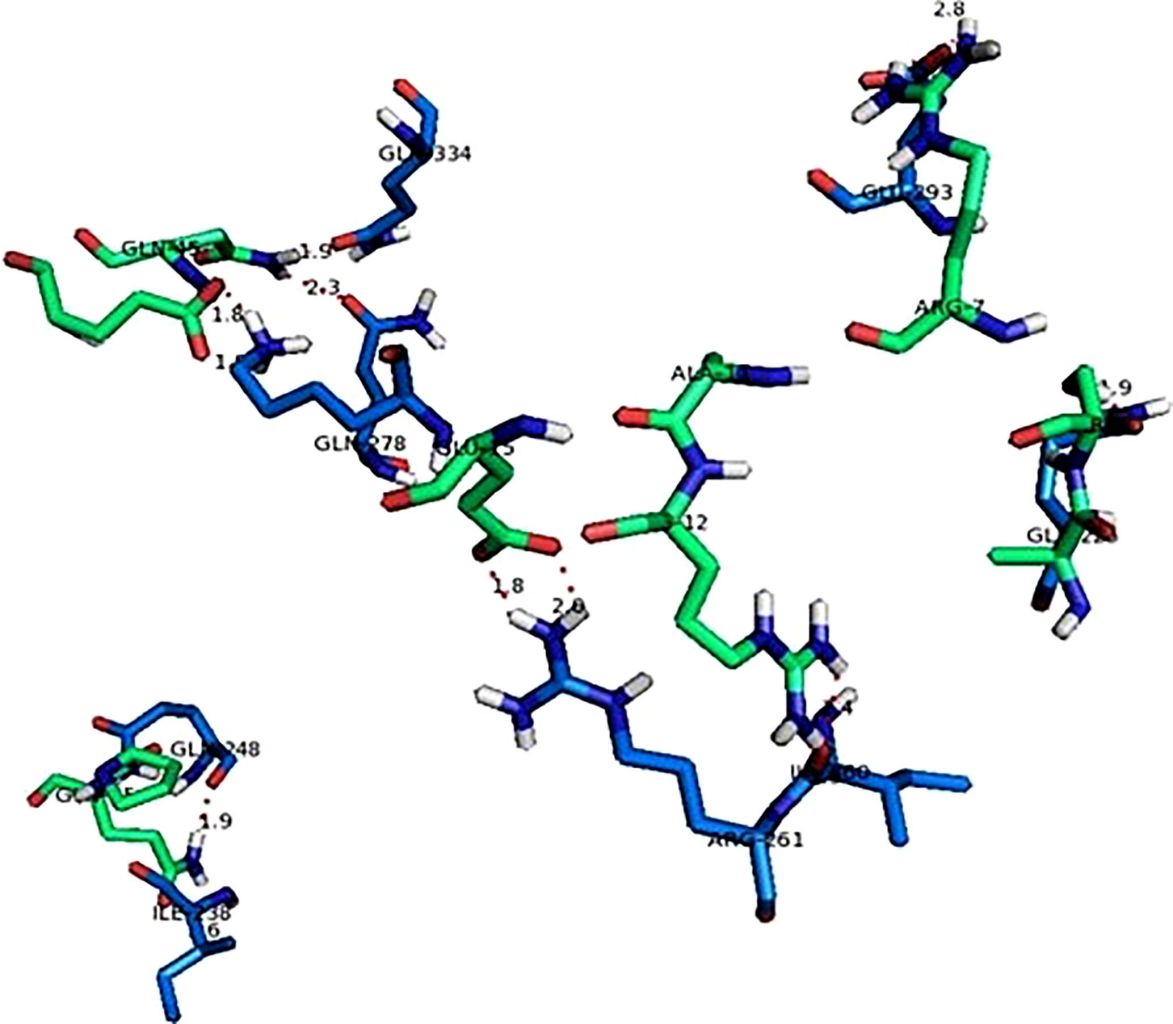
Interaction between human NLRP3 (Green sticks) and viral fusion protein (Blue sticks).

The protein-protein interactions between viral fusion protein and human NLRP3 protein are shown in **Figure 8**. The figure showed below exhibits only polar contacts between the proteins. The most significant interaction in this was between the majority of glutamine residues of both receptor and ligand proteins. There were a total of 11 polar contact points between the NLRP3 and viral fusion protein. All of the contact point interaction distances are within the acceptable range (4 Å) which indicates the strong bonding between the proteins as shown in **Table 5**.

**Table 5:** Receptor and ligand amino acid interactions and distances.

Serial No.	Amino acid (Receptor protein)	Amino acid (Ligand protein)	Distance in Angstrom	Type of interaction
**1**	GLN-35	GLN-248	1.9	Polar
**2**	GLN-35	ILE-238	2.6	Polar
**3**	GLN-45	GLN-278	2.3	Polar
**4**	GLN-45	GLN-334	1.9	Polar
**5**	SER-5	GLN-225	1.9	Polar
**6**	GLU-293	ARG-7	1.9, 2.8	Polar
**7**	GLU-15	ARG-261	1.8, 2.0	Polar
**8**	ARG-12	ILE-260	1.9, 2.4	Polar

### 3.3 NPs optimization by BoxBehnken factorial design

**Figure 9:**
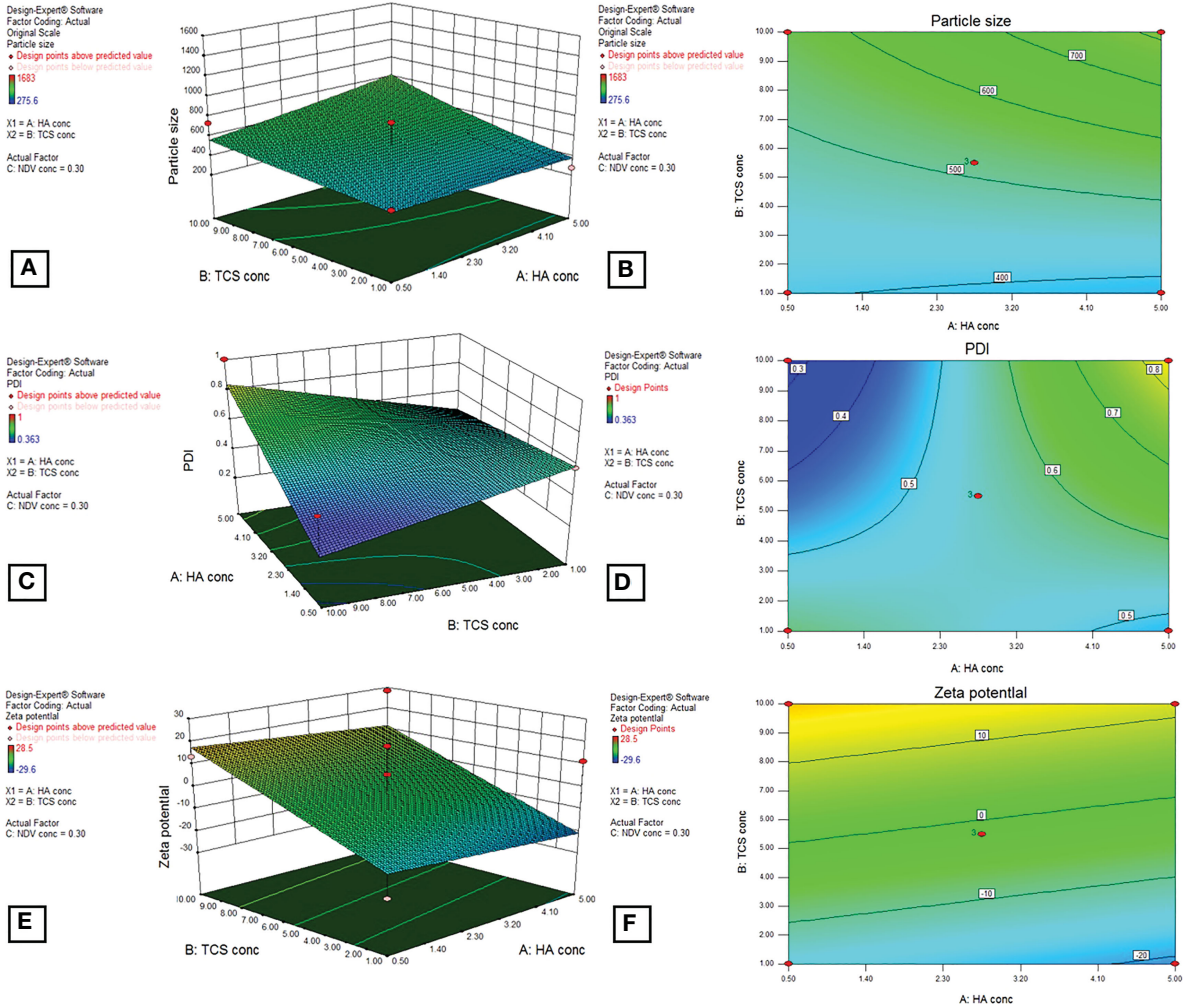
3D graphs by BoxBehnken factorial design for dependent variables, particle size **(A, B)**, PDI **(C, D)**, and zeta potential **(E, F)** by using DOE outputs for the optimization of HA-coated NDV-loaded NF.

For optimization of HA coated NDV loaded nanoparticles, the dependant variables that included size, PDI and zeta potential were mesured using design of expert (DOE) version 8.0.6.1 as shown in **Table 2**. The mentioned parameters were displayed by BoxBehnken factorial design for selected nanoformulation (**Figure 9**).

### 3.4 Preparation of HA-ThCs-NDV NPs

The virus loaded, CD44 targeted nanoparticles were formulated by ionic gelation method. Using TPP as crosslinker. These nanoparticles were formed based on electrostatic forces of attraction between cationic group (NH_2_+) of thiolated chitosan and carboxylic group (COOH) of hyaluronic acid. The prepared formulation was then analyzed for various physiochemical and morphological characteristics (12).

### 3.5 Physiochemical and morphological analysis

**Figure 10:**
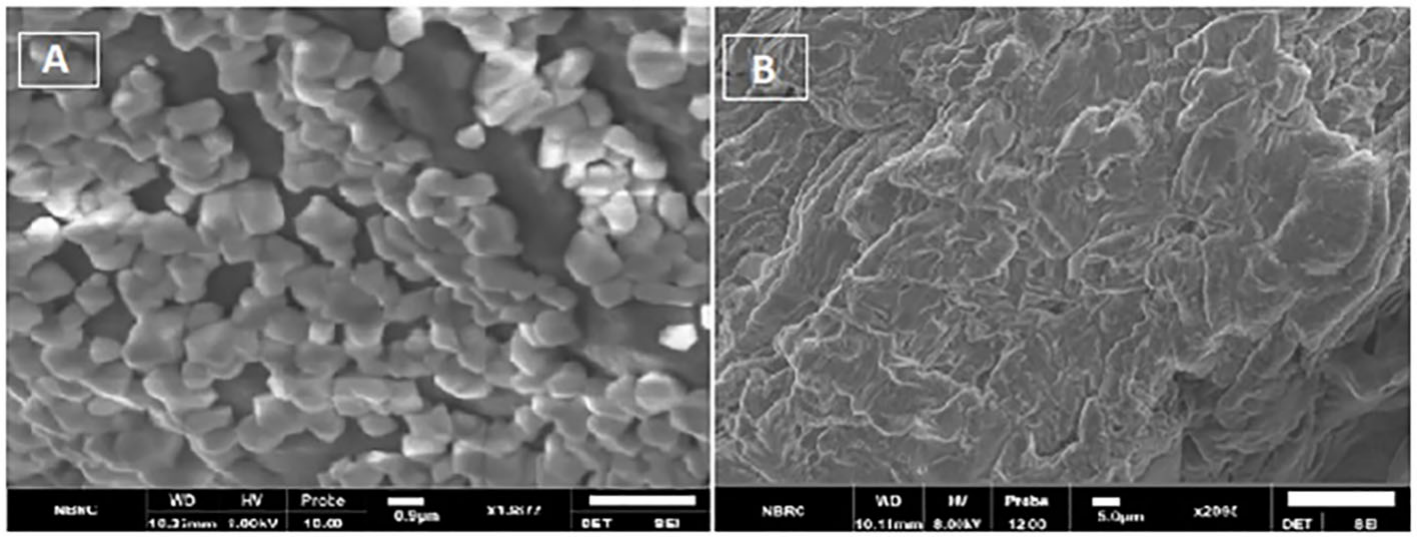
**(A)** SEM images showing spherical features of HA-ThCs-NDV NPs, (B) surface morphology of NFs.

**Figure 11:**
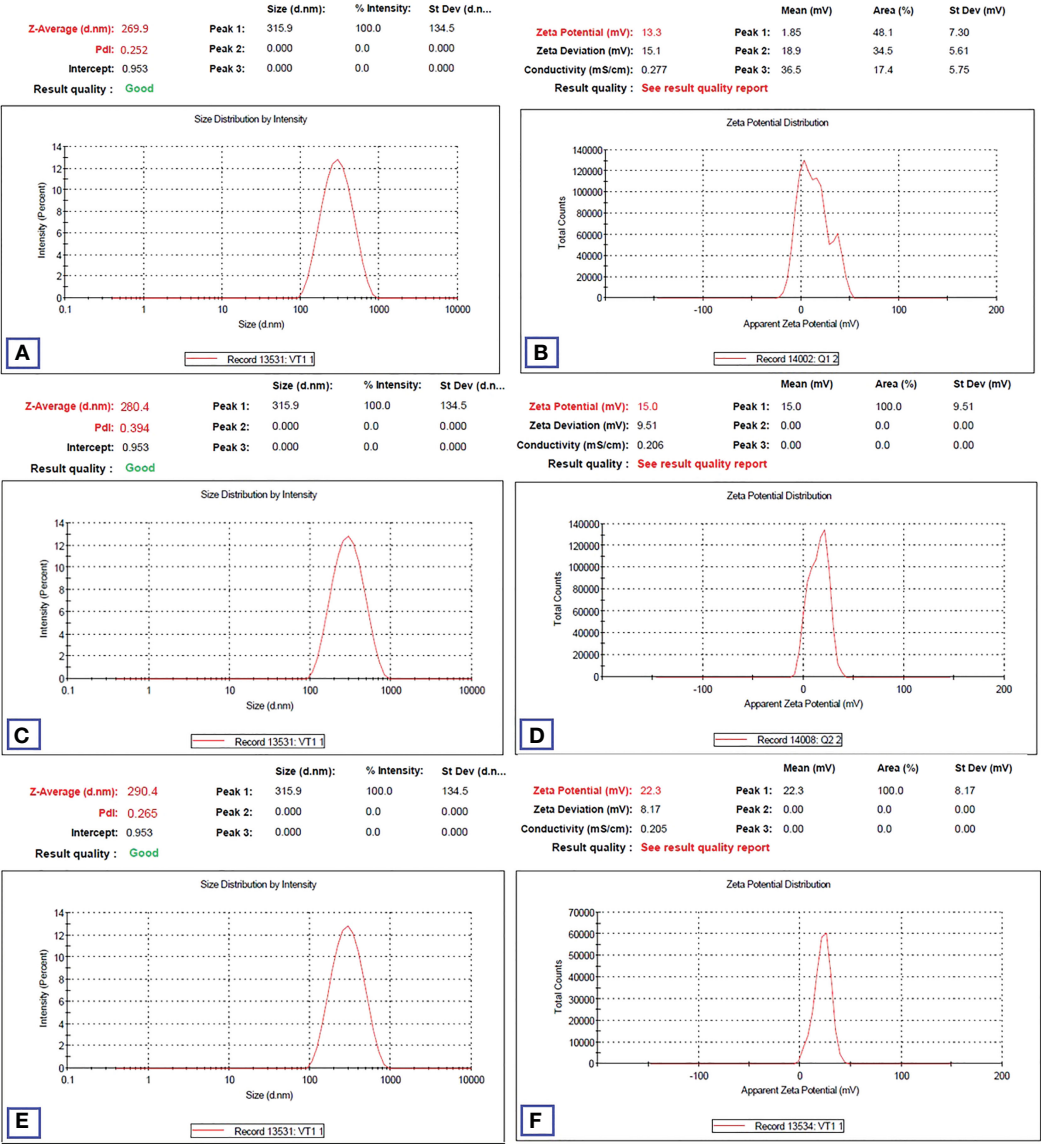
Particle size, zeta potential and PDI values (n=5) for blank NFs **(A, B)**, NDV-loaded ThCs **(C, D)**, and HA-coated NDV-loaded ThCs-NF **(E, F)**.

HA-TCs-NDV NPs were extensively characterized using zeta sizer, which revealed the size, charge, and the polydispersity index (PDI) of nanoparticles. Zeta analysis showed even distribution of nanoparticles as seen in **Figure 10**. The minimum particle size of ThCs NPs was 269.9nm with 13.3 mV zeta potential and 0.25 PDI (**Figures 11A, B**). The analysis of NDV loaded in thiolated chitosan (TCs-NDV) revealed a slight increase in NPs size, which was 280.4nm with 15 mV zeta potential and 0.394 PDI (**Figures 11C, D**). The NDV loaded ThCs (HA-ThCs-NDV) NPs exhibited 290.4nm average particle size with 22.3 mV zeta potential and 0.265 PDI (**Figures 11E, F**), at the polymer (ThCs) concentration of 1.0 mg/ml, HA at 0.5 mg/ml, with a half dose (not less than 500 TCID units) of NDV.

**Figure 12:**
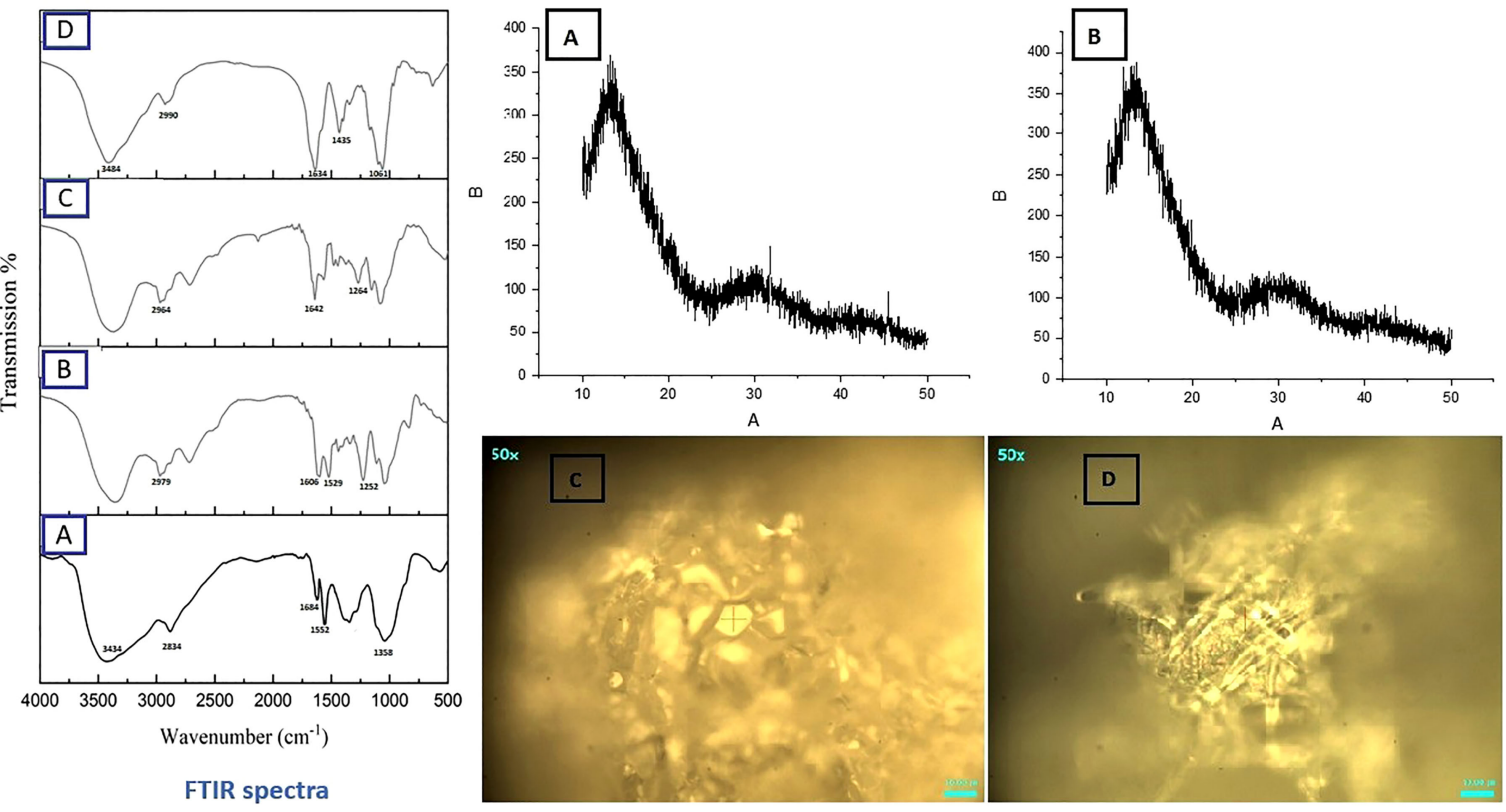
FTIR Spectrum (Left) of ThCs **(A)**, Blank HA-ThCs NFs **(B)** ThCs-NDV **(C)**, and HA-ThCs-NDV NFs **(D)**. XRD (Top Right) ThCs-NDV **(A)**, and HA-ThCs-NDV **(B)**, and Raman analysis (Lower Right), ThCs-NDV **(C)**, and HA-ThCs-NDV **(D)**.

SEM analysis revealed smooth surface and spherical features of nanoparticles (**Figure 10**). Spectra from FTIR indicated deflection at around 3430 to 3403 cm^-1^ owing to presence of OH functional group in ThCs band, at 2901 to 2885 cm^-1^ due to stretching of CH, at 1635 to 1606 cm^-1^ due to stretching of amide C=O in the nanoformulations. An inclined stretching peak observed at 2496 cm^-1^ confirmed the successful thiolation of chitosan (**Figure 12** Left). Raman analysis showed highest peak at 600cm^-1^ owing to bending vibration of C-C-O bonds while the consistent decline was observed at approximately 950cm^-1^ (**Figure 12** right **C, D**). XRD analysis revealed that virus enclosed nano-formulation is crystalline in nature The prominent reflection was observed at 2θ = 14.8° in both nanoformulations, and significantly less reflection was seen at 21.5° in NDV loaded nanofomulation. No significant change in peaks was observed in blank and virus loaded nanoformulation as these outcomes show the degree of crystallinity of NDV nanoformulation due to the hydrogen bonding present in intermolecular and extra molecular forces, which may have been retained in virus loaded formulation compared with blank (**Figure 12** right **A, B**).

### 3.6 Viral quantification of NDV nanoparticles

#### 3.6.1 Tissue culture infective dose (TCID_50_)

In order to calculate number of infectious particles encapsulated in nanoparticles, TCID_50_ was calculated by Reed and Munich method, in comparison with commercially available NDV in HeLa cells at 7 p.i.d. TCID_50_, also called endpoint dilution, represents the dilution that infected 50% of infected cells. On 7 p.i.d, TCID_50_ was 31.6x 10^4^ TCID_50_/mL for pure NDV, 2.5x 10^5^ TCID_50_/mL for ThCs-NDV and 2.63x 10^6^ TCID_50_/mL for HA-ThCs-NDV TCID_50_.

#### 3.6.2 Cytopathic effects (CPE)

**Figure 13:**
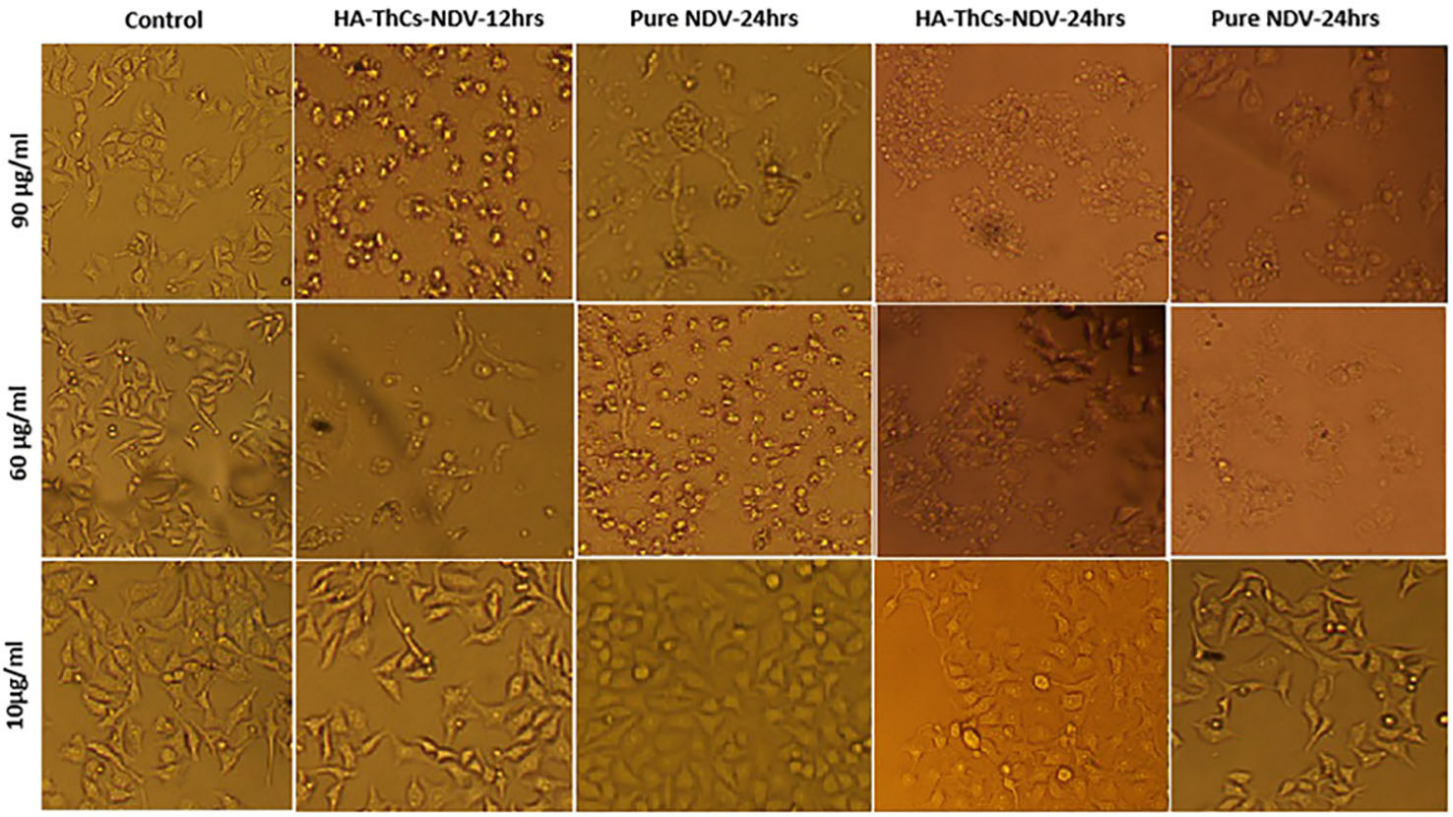
Change in morphology of treated HeLa cells with pure NDV and HA-ThCs-NDV in time and dose-dependent manner.

For evaluating the cytopathic effect of NDV-loaded nanoparticles in comparison with pure NDV, HeLa cells were treated with pure NDV and HA-ThCs-NDV NPs at different concentrations. The minimum cytopathic effect was observed in cells treated with 10 µg/ml while highest cytopathic effect was observed at concentration of 90 µg/ml. The cytopathic effect was marked by morphological changes such as rounding, clumping (syncytia formation) and detachment of cells from well plate as shown in **Figure 13**. The oncolytic effect exhibited in form of apoptosis confirmed that HA-ThCs-NDV NPs efficiently delivered NDV inside HeLa cells without compromising their oncolytic potential.

### 3.7 *In vitro* NDV release from NFs

**Figure 14:**
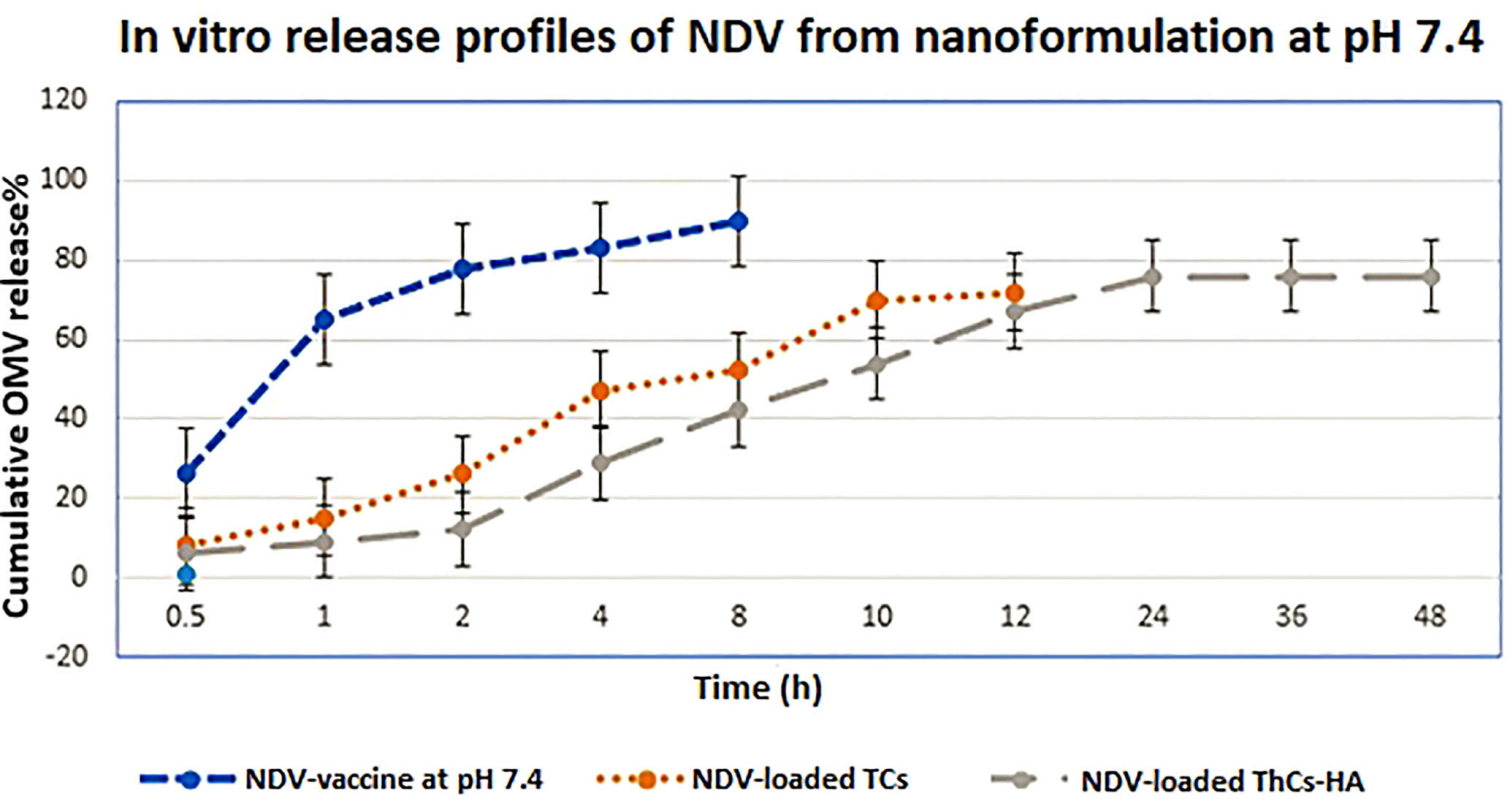
*In vitro* release profiles of virus from NDV-loaded NFs in PBS at pH 6.8. Mean values were analyzed using the student’s test (n=3, mean ± SD, p ≤ 0.05).

The release of NDV from nanoformulation occurred till 48 hours, which confirms the sustained release NDV from nanoformulation as compared to pure NDV. The experiment was repeated three times and all results were obtained in triplicates as shown in **Figure 14** below.

### 3.8 *In vitro* anticancer activity of NDV NFs

**Figure 15:**
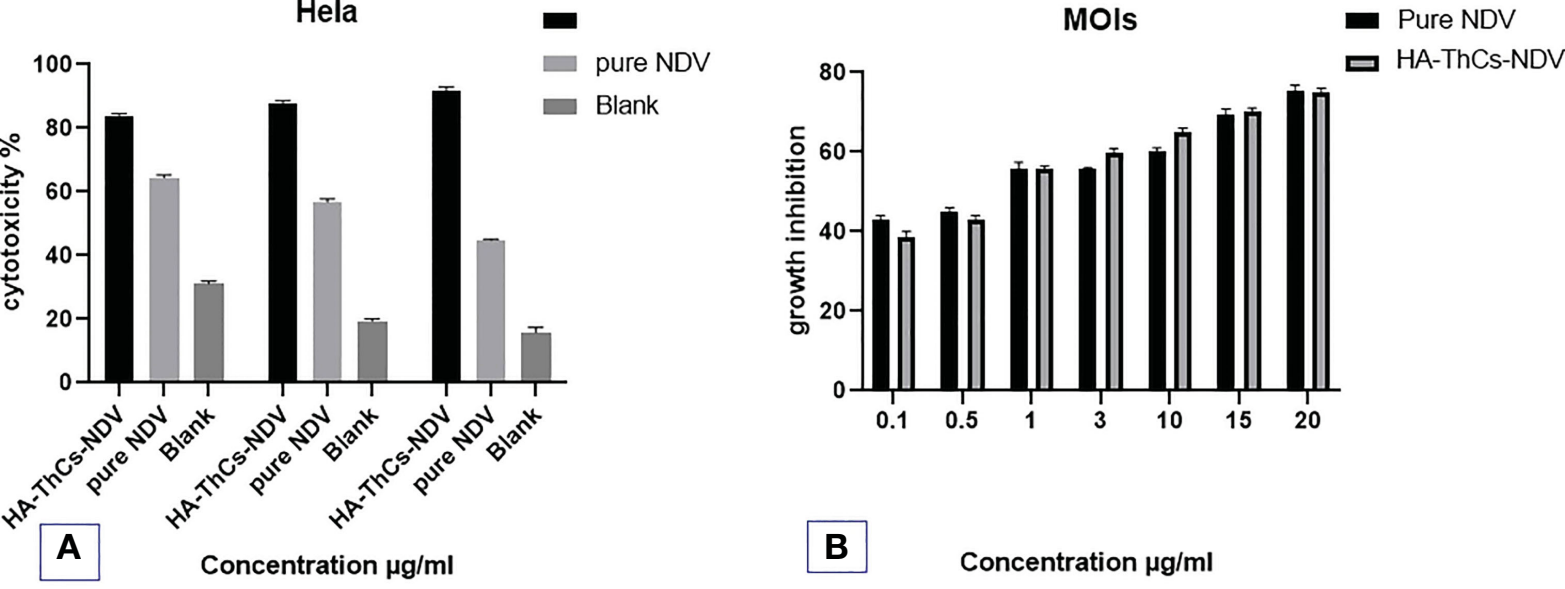
*In vitro* anti-cancer activity of the Pure NDV and HA-ThCs-NDV NFs on HeLa cells for TCID_50_. **(A)** Cytotoxicity potential of pure NDV and NDV NFs in dose dependent manner. **(B)** The growth inhibition at different MOIs of pure NDV and NDV NFs (n=3, mean ± SD, p ≤ 0.05).

HeLa cells were inoculated with NDV encapsulated NFs at various MOIs (0.6, 1, 5, 8, 12,18, 20) and cytopathic effect was determined for calculation of TCID_50_ (tissue culture inhibitory dose). Following inoculation and incubation time of 72 hours, growth inhibition and TCID_50_ was measured using MTT colorimetric assay. MTT analysis revealed the oncolytic potential of NFs encapsulated NDV in treated and control cells. The outcomes showed that pure NDV and HA-ThCs-NDV have oncolytic activity with IC_50_ of 5.6 and 3.8 viral titer respectively on HeLa cells. The oncolytic potential increased with increase in MOI of virus as shown in **Figure 15**.

### 3.9 Stability parameters

**Figure 16:**
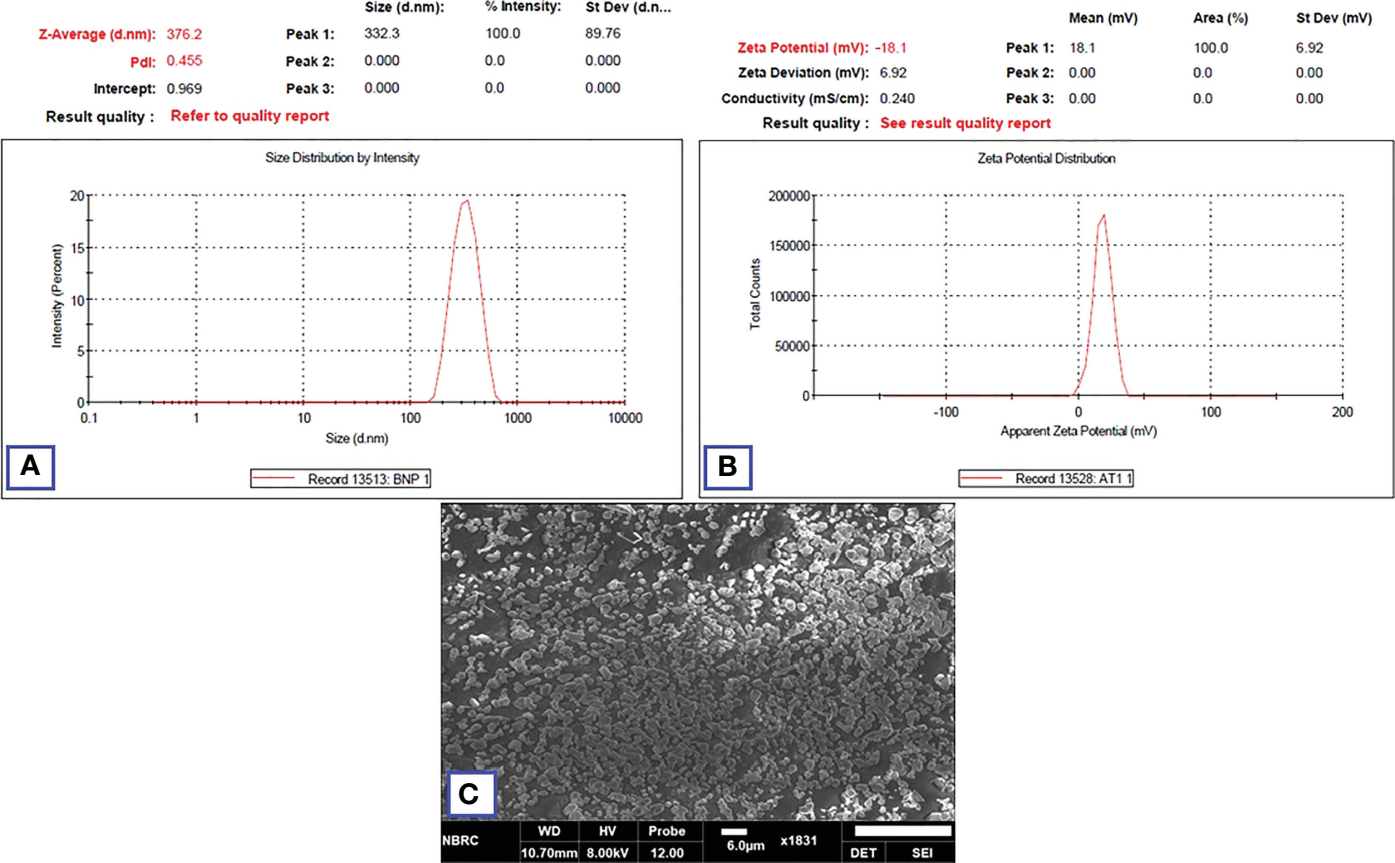
Zeta analysis of nano-formulation showing the size **(A)**, zeta potential **(B)** after three months of storage. SEM image of lyophilized NFs after 3 months **(C)**.

The stability parameters were analyzed for NFs (HA-ThCs-NDV) by evaluating changes in particle morphology, size, zeta potential and PDI after 3months, while the nano-formulation was stored at an ambient temperature of 37°C and refrigerated at 4°C. The results showed that formulation stored in liquid form showed increase in particle size from 290.4nm to 376.2nm, change in zeta potential from 22.3 mV to -18.1mV and PDI from 0.265 to 0.455. While, the formulation stored in lyophilized form was stable after 3 months and did not exhibit any change as shown in **Figure 16** below. Thus, virus encapsulated nanoparticles are stabilized in lyophilized powder form for long-term storage use.

## 4 Discussion

The paramyxovirus, NDV causes fatal respiratory diseases in birds but has a relevant good safety profile in humans. It induces mild fever or conjunctivitis in humans that does not last long., Overall human population is seronegative for antibodies against NDV. In neurooncology, preclinical in- vitro and in-vivo studies reported that oncolytic NDV acts as effective *in situ* tumor vaccine by synergistic anti-inflammatory tumor response with virus induced selective cancer cell lysis, thereby significantly multiplying the multitude of antitumor response in glioblastoma (30). In lung cancer, an attenuated lentogenic isolate of Newcastle disease virus (NDV), strain FMW (NDV-FMW) induced caspase-dependent apoptosis in lung cancer spheroids and triggered autophagic degradation by inhibition of the AKT/mTOR pathway. Due to defective type I interferon (IFN) signaling against intracellular viruses in cancer cells, NDV infects and multiply in cancer cells, whereas healthy cells neutralize the viral invasion due to efficient intracellular antiviral IFN response (31).

Human body has high tolerability against NDV as the virus does not have a mechanism to evade from innate immune response in humans. One of the biggest concerns in oncolytic virotherapy is host immunity that mediates viral clearance through complement mediated antibody-dependent neutralization. Therefore, a biocompatible, polymer based targeted drug delivery system with ability of sustained release can enhance oncolytic potential and prolong availability of virus in tumor microenvironment (32). As reported, mesenchymal cells encapsulating NDV for selective delivery to murine TC-1 cells and in tumor models showed significantly enhanced oncolytic effect as compared to naked virus (6). NDV encapsulated in thiolated chitosan not only prevented virus from immune neutralization but the sustained release profile for up to 48 hours, thus extending the exposure of tumor cells to the virus. Moreover, the targeting capacity added by CD44 targeting HA enhanced the attachment and retention of these nanoparticles at the tumor site. As reported earlier, a CD44 drug delivery system based on thiolated chitosan, surface functionalized with hyaluronic acid proved to be an ideal approach for delivery of oncolytic measles virus to prostate cancer tumors. This drug delivery system provides sustained release of virus for approximately 48 hours (12). Increased sensitivity of HA to CD44 help these active targeting of the NPs to accumulate inside tumor and therefore provide a promising antitumor response. In another study, active targeting of CD44 expression in breast cancer by doxorubicin and cisplatin co-loaded chitosan-HA nanoparticles provided significant anticancer activity (33).

Bioinformatic analysis has become the first step towards development of targeted drug delivery systems. Before preparing CD44 targeted nanoformulation against HeLa cells, the expression of CD44 on HeLa cells was analyzed by using data from Human Protein Atlas data repository for immunoreactivity of CD44 antibody HPA005785 with HeLa cells. To support our strategy of choosing CD44 as a potential receptor for effective antitumor targeting in cervical cancer specifically, the role of CD44 expression in cervical cancer in overall survival and disease-free survival of patients was also analyzed. As reported, folate receptor targeting by polyethylene glycol probe for 5-Fluorouracil was evaluated using bioinformatic analysis like molecular docking studies and MD simulation prior to preparation of the nanoconjugates (34). The efficacy of various pharmacological compounds was studied against CCND1(human D-type cyclin gene)/CDK4 (Cyclin-dependent kinase 4)/PLK1 (polo like kinase 1)/CD44 using various bioinformatic tools and the results showed CD44 as a highly promising antitumor target in different solid tumors (35). *In silico* analysis for virus-host protein interactions was performed to evaluate the feasibility of NDV interaction with intracellular targets in host. In our study, the host and viral protein-protein docking studies showed the most significant interaction was between viral hemagglutinin neuraminidase with TNF and viral fusion protein with NLRP3. There was a total of 18 polar contact points between the proteins which included both ionic interactions and hydrogen bonding. The activation of TNF-α, a proinflammatory cytokine promotes activation of signaling cascade leading to cell necrosis or apoptosis. On the other hand, NLRP3 inflammosome is major mediator of innate immune response activation which promotes caspase-1 activation.

The nanoformulation for NDV encapsulation was prepared using green synthesis approach through ionotropic gelation method which involves attraction between oppositely charged molecules (NH_2_ of chitosan and COOH of hyaluronic acid). In this study we used green synthesis approach that do not no require harsh chemicals, undesired by products and acids/alkalis during preparation procedure (12). Morphological and physiochemical characterization of HA-ThCs-NDV revealed that the nanoparticle size was 290.4nm, zeta potential was 22.3 mV with 0.265 PDI. These results are in accordance with our earlier study that reported NP size of 275.6 mm, ± 11.5mV zeta potential and PDI = 0.372 for targeted delivery of oncolytic measles virus loaded into polymeric thiolated chitosan nanoparticles and targeted against CD44 receptors in prostate cancer cells (36). Another study reported encapsulation of NDV in chitosan nanoparticles with particle size of 371nm and a zeta potential of +2.84 mV (37). Furthermore, mesoporous silica nanoparticles were used as a delivery carrier for encapsulation of NDV, the nanoparticles had spherical shape with zeta potential of 17.4 ± 1.7 mV and average particle size of 243 nm. Particle size under 300nm is considered favorable as delivery vehicles as the particles greater than 300 nm are cleared from systemic circulation through reticuloendothelial system (23, 38).

Cytopathic effect of HA-ThCs-NDV in comparison with pure NDV was analyzed by evaluating changes in cell morphology at different time intervals and at different doses. Rounding, aggregation and syncytia formation are the hallmarks of the oncolytic virus which were clearly marked in the current study (**Figure 14**). According to our findings,TCID_50_ on 7 p.i.d 31.6x 10^4^ TCID_50_/mL for pure NDV, 2.5x 10^5^ TCID_50_/mL for ThCs-NDV and 2.63x 10^6^ TCID_50_/mL for HA-ThCs-NDV. These findings depicts enhanced uptake and infectivity of ThCs-NDV owing to mucoadhesive properties of the thiolated chitosan and an even increased infectivity when targeting capacity was added. The IC_50_ value at MOIs (0.6, 1, 5, 8, 12, 18, 20) was 5.6 and 3.8 for pure NDV Lasota virus and HA-ThCs-NDV respectively after incubation period of 72 hours. These findings suggest that CD44 receptor mediated uptake of HA-ThCs-NDV produced a greater oncolytic effect in HeLa cells as compared to naked NDV virus. Another study reported similar high cytopathic effect of HA functionalized thiolated chitosan encapsulated oncolytic measles virus against prostate cancer cells with IC_50_ of 5.1 and 3.52 for pure measles and NPs loaded NDV (36).

## 5 Conclusion

The current research was conducted to achieve sustained release of NDV by encapsulation in biocompatible polymer thiolated chitosan. Active targeting against CD44 receptor was achieved by surface functionalization with hyaluronic acid. Physiochemical characterization confirmed the desirable particle size with a suitable charge for enhanced uptake by cancer cells in acidic pH of the tumor microenvironment. Sustained release of the virus for up to 48 hours is promising approach as it will enhance the bioavailability of oncolytic virus in tumor microenvironment. As compared to pure NDV, these NFs exhibited excellent growth inhibition at low doses, the inhibitory effect was dose dependent (p<0.05). All of these characteristics enhance the efficacy, targeting and anticancer outcome of NDV NPs, hence supporting this nanoformulation as an effective immunomodulator therapy. These findings will be further evaluated through *in vitro* experiments and *in vivo* cancer models.

## Data availability statement

The original contributions presented in the study are included in the article/supplementary material. Further inquiries can be directed to the corresponding authors.

## Author contributions

KK conducted major study including designing and execution of experiments, analysis and investigation. FN provided support in nanoparticle preparation and characterization. MA contributed in data analysis and interpretation of results, methodology and funding acquisition. SA and TA proofread this article and supervised this project. All authors read and approved the final manuscript.
