# Corrigendum: CD44 targeted delivery of oncolytic Newcastle disease virus encapsulated in thiolated chitosan for sustained release in cervical cancer: a targeted immunotherapy approach

**DOI:** 10.3389/fimmu.2024.1391710

**Published:** 2024-03-11

**Authors:** Kousain Kousar, Faiza Naseer, Maisa Siddiq Abduh, Sadia Anjum, Tahir Ahmad

**Affiliations:** ^1^Industrial Biotechnology, Atta-Ur-Rahman School of Applied Biosciences, National University of Sciences and Technology, Islamabad, Pakistan; ^2^Shifa College of Pharmaceutical Sciences, Shifa Tameer e Millat University, Islamabad, Pakistan; ^3^Immune Responses in Different Diseases Research Group, Department of Medical Laboratory Sciences, Faculty of Applied Medical Sciences, King Abdulaziz University, Jeddah, Saudi Arabia; ^4^Center of Excellence in Genomic Medicine Research, King Abdulaziz University, Jeddah, Saudi Arabia; ^5^Department of Biology, University of Hail, Hail, Saudi Arabia

**Keywords:** oncolytic Newcastle disease virus, cervical cancer, green synthesis, sustained release, CD44, polymeric nanoparticles

In the published article, there was an error in **Figure 10** (C, D) TEM images of HA-ThCs-NDV NFs at 200nm and 500nm respectively and **Figure 13** Change in morphology of treated HeLa cells with pure NDV and HA-ThCs-NDV in time and dose-dependent manner as published.

1- The image in **Figure 10** (C, D) part highly resembles/overlaps with image of same nanoformulation from our own previous publication in Frontiers in Pharmacology, that occurred due to wrong tagging during data analysis (https://doi.org/10.3389/fphar.2022.1073004) - Figure 7B.

2- The image in **Figure 13** of panel 10 ug/ml of pure NDV-24 hrs and HA-ThCs-NDV-24 hrs is incorrect as wrong slide was provided and the image highly resembles the image from our previous publication in Frontiers in Pharmacology, (https://doi.org/10.3389/fphar.2022.1073004) - and Figure 14a. The corrected **Figures 10** and **13** and their captions appear below.

**Figure 10:**
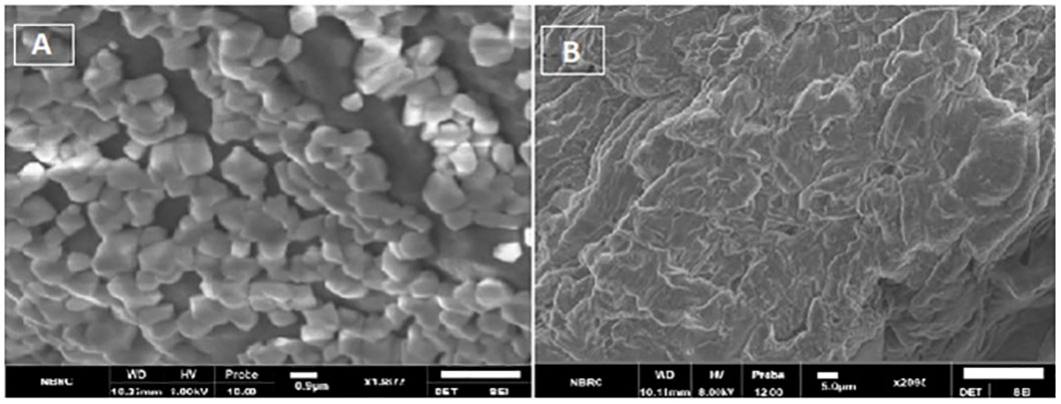
**(A)** SEM images showing spherical features of HA-ThCs-NDV NPs, **(B)** surface morphology of NFs.

**Figure 13:**
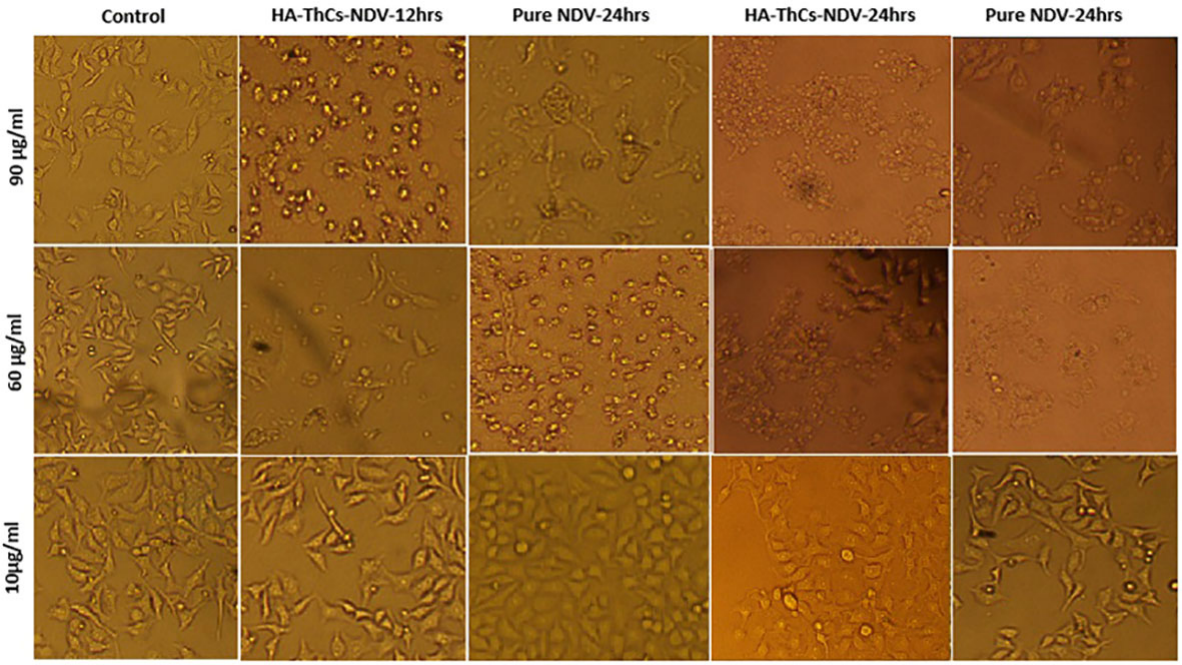
Change in morphology of treated HeLa cells with pure NDV and HA-ThCs-NDV in time and dose-dependent manner.

The authors apologize for this error and state that this does not change the scientific conclusions of the article in any way. This error occurred as we were working on both studies (https://doi.org/10.3389/fphar.2022.1073004 and https://doi.org/10.3389/fimmu.2023.1175535) simultaneously and some of the data/slides were wrongly tagged by students working on the project.

